# Fast-Processable Non-Flammable Phthalonitrile-Modified Novolac/Carbon and Glass Fiber Composites

**DOI:** 10.3390/polym14224975

**Published:** 2022-11-17

**Authors:** Daria Poliakova, Oleg Morozov, Yakov Lipatov, Alexander Babkin, Alexey Kepman, Viktor Avdeev, Boris Bulgakov

**Affiliations:** Division of Chemical Technology and New Materials, Department of Chemistry, M.V. Lomonosov Moscow State University, Leninskie Gory St, 1-11, 119991 Moscow, Russia

**Keywords:** phthalonitrile, novolac, heat-resistance, mechanical properties, non-flammable, composite

## Abstract

Phthalonitrile resins (PN) are known for their incredible heat resistance and at the same time poor processability. Common curing cycle of the PN includes dozens hours of heating at temperatures up to 375 °C. This work was aimed at reducing processing time of phthalonitrile resin, and with this purpose, a novolac oligomer with hydroxyl groups fully substituted by phthalonitrile moieties was synthesized with a quantitative yield. Formation of the reaction byproducts was investigated depending on the synthesis conditions. The product was characterized by ^1^H NMR and FT-IR. Curing of the resins with the addition of different amounts of novolac phenolic as curing agent (25, 50 and 75 wt.%) was studied by rheological and DSC experiments. Based on these data, a curing program was developed for the further thermosets’ investigation: hot-pressing at 220 °C and 1.7 MPa for 20 min. TGA showed the highest thermal stability of the resin with 25 wt.% of novolac (T_5%_ = 430 °C). The post-curing program was developed by the use of DMA with different heating rates and holding for various times at 280 or 300 °C (heating rate 0.5 °C/min). Carbon and glass fiber plastic laminates were fabricated via hot-pressing of prepregs with T_g_’s above 300 °C. Microcracks were formed in the CFRP, but void-free GFRP were fabricated and demonstrated superior mechanical properties (ILSS up to 86 MPa; compressive strength up to 620 MPa; flexural strength up to 946 MPa). Finally, flammability tests showed that the composite was extinguished in less than 5 s after the flame source was removed, so the material can be classified as V-0 according to the UL94 ratings. For the first time, fast-curing phthalonitrile prepregs were presented. The hot-pressing cycle of 20 min with 150 min free-standing post-curing yielded composites with the unique properties. The combination of mechanical properties, scale-up suitable fast-processing and inflammability makes the presented materials prospective for applications in the electric vehicle industries, fast train construction and the aerospace industry.

## 1. Introduction

Fiber-reinforced polymer composites have attracted the attention of scientists and engineers working in different industries requiring high-performance materials and light-weight constructions. Composites help reduce the weight of fast-moving vehicles in the aerospace, automotive and railroad industries, decreasing fuel consumption and CO_2_ emissions. Due to their high rigidity and specific strength, fiber-reinforced composites are considered as alternatives to metals. Metalwork for complex-shaped parts is a time and resource consuming process, while computer simulations of composite properties and processing [1,2,3,4,5,6] facilitate design and production of composite parts, expanding their applications in different fields. Nowadays, composite parts give not only the benefits of lightweight and rigid structures but also provide advantages in the design of complex-shaped parts, product assembly and long-term operation due to high weather, chemical and fatigue resistance [7]. One of the critical limitations of composites is their operating temperature due to the polymer nature of the matrix. In the aerospace industries, the most commonly used polymer matrix is epoxy, but it can operate at temperatures up to 200 °C [8].

At the same time, most of the known heat-resistant thermosetting resins, including addition-cured phenolics [9,10,11,12], benzoxazines [13,14,15], cyanate esters [16,17,18] and bismaleimides [19,20,21,22,23,24], can be operated at temperatures only up to 270 °C. Because of their high degradation temperatures (T_5%_) over 500 °C and T_g_’s of the thermosets exceeding 400 °C [25], phthalonitrile resins are extremely attractive as matrices for fiber-reinforced composites designed for operation at elevated temperatures.

Since the early reports describing composites with phthalonitrile matrices [26,27], the most common way of composite fabrication was solution-impregnated prepreg consolidation [28,29,30,31,32,33], which was caused by high melting temperatures of the monomers. The studies aimed to synthesis of the low-melting phthalonitriles and the use of reactive plasticizers [34,35,36,37,38,39,40,41] enhanced processability of the resins and made them suitable for cost-effective processing methods, such as vacuum infusion [42,43,44,45], RTM [46] and dry prepreg molding [43]. On the other hand, mass production of composite parts requires fast processing methods of composite fabrication, such as hot-pressing. Curing of phthalonitriles occurs with formation of isoindoline, phthalocyanine and triazine moieties [25] and usually takes hours at 300–400 °C [47,48,49,50,51,52,53,54]. There are published studies aimed to improve phthalonitrile curing parameters by introducing new curing agents [48,55,56,57,58], but there were no reports stating significant reduction of the curing time. Phthalonitrile composites are known for their great heat resistance and high LOI values of composites exceeding 80% [44,46] and low ingnitability [59], which makes them prospective materials for fire resistant walls in transport vehicles and engines. At the same time no data describing in-flame tests of phthalonitrile composites were published. 

In the present study, we suggested that phthalonitrile-modified novolac resin (PNN) can be fast-cured since it has a high content of phthalonitrile groups bonded to novolac oligomers providing possibilities for high cross-linking density and viscosity proper for hot-pressing. From the other hand, in the previous reports describing phthalonitrile-modified novolac, curing times were 6–20 h [60,61,62,63], which was not shorter than typical phthalonitrile curing times. In [61] Zhang et al. used novolac resin in concentrations up to 10% as a curing agent for phthalonitrile-modified novolac oligomers with partial substitution with the aim to improve the mechanical performance of fiber-reinforced composites and decreasing curing time down to 47 min at 170 °C. In this work, we decided to use novolac resin as a curing agent in higher concentrations to promote fast curing of phthalonitrile-modified novolac resin and provide good miscibility of the oligomers of the resin. To better estimate the influence of hydroxyl concentration on the curing process and maintain reproducibility of the processing, novolac with fully substituted hydroxyl groups by phthalonitrile rings was taken as a thermosetting oligomer.

This approach helped us to reduce the curing cycle of the resin down to 20 min and using hot-pressing technology to fabricate composites demonstrated excellent fire retardant and high mechanical properties at elevated temperatures.

## 2. Materials and Methods

### 2.1. Materials

All manipulations with the oxidation- and moisture-sensitive compounds were carried out under argon atmosphere. Acetone, methylethylketone (MEK) *N*,*N*-dimethylacetamide (DMAc), potassium carbonate were purchased from Chimmed (Moscow, Russia), 4-nitrophthalonitrile was purchased from Central Drug House (New Delhi, India), novolac resin of 99.7% purity was purchased from Metadynea (Moscow, Russia) under trade name SF-0112a and was dried before use on a rotary evaporator at 110 °C using a nitrogen trap. Carbon fabric 22,502 from JSC INCMaT (Moscow, Russia) 2 × 2 twill weave 3k UMT42S from Umatex (Moscow, Russia) was used for CFRP preparation. Glass fabric T-10P-14 from JSC Steclonit (Ufa, Russia) 8 × 3 satin weave was used for GFRP preparation. 

### 2.2. Characterization

Nuclear magnetic resonance (NMR) spectra were recorded on a Bruker Avance II 600 (Billerica, MA, USA) at 600 MHz for ^1^H. Fourier transform infrared (FT-IR) spectra were recorded on a Bruker Tensor-27 spectrophotometer (Billerica, MA, USA) in the range of 4000–400 cm^−1^ using KBr pellets. Thermal stability of blends was evaluated via thermogravimetric analysis (TGA) on a Netzsch TG 209 P3 Tarsus (Selb, Germany), at heating rate of 10 °C/min in range of 40–900 °C in nitrogen purge of 50 mL/min. Differential scanning calorimetry (DSC) was performed on a Netzsch DSC214 Polyma (Selb, Germany) at a heating rate of 10 °C/min. Rheological behavior was studied with an Anton Paar MCR 302 rheometer (Graz, Austria) in the temperature range from 110–250 °C at a heating rate of 2 °C/min in oscillation mode. Elemental analysis was performed using Perkin Elmer 2400 Series II CHNS/O Elemental Analyzer (Waltham, MA, USA) at the Laboratory of Microanalysis of INEOS RAS, Moscow. Microphotographs were obtained using a scanning electron microscope (SEM) TESCAN VEGA3 LMU (Brno, Czech Republic) at an accelerating voltage of 20 kV. To obtain images, the samples were poured with epoxy resin into a special mold, then cut, ground and polished. The surfaces of the samples were sputter-coated with gold for better resolution. Interlaminar shear strength was measured with a Tinius Olsen 50ST (Redhill, UK) according to GOST 32659-2014 at room temperature, 200 °C and 250 °C (7 samples, 20 × 10 mm). Compression strength and elasticity modulus were measured with an Instron 5985 (Norwood, MA, USA) according to GOST 56812 at room temperature (7 samples, 60 × 15 mm). Tensile strength and elasticity modulus were measured with an Instron 5985 (Norwood, MA, USA) according to GOST 56785 at room temperature. Dynamic mechanical analysis (DMA) was performed on a TA Instruments DMA Q800 (New Castle, DE, USA) by scanning the specimens (55 mm × 5 mm × 2 mm) over temperature of 50–450 °C with frequency of 1 Hz and under N_2_ atmosphere. Samples were cut in the direction of 45 degrees.

### 2.3. Synthesis of Phenol-Containing Phthalonitrile Oligomer (PNN) Solution

Novolac resin (106 g) and DMAc (300 g) were added to a 1 L three-necked round-bottom flask equipped with a reflux condenser and stirred under an argon atmosphere. After complete dissolution of the novolac resin, anhydrous potassium carbonate (151.8 g, 1.1 mol) was added and the mixture was stirred for 1 h at 40 °C. 4-Nitrophthalonitrile (173 g, 1 mol) was added to the reaction mixture and stirring was continued for 20 h at 40 °C. The progress of the reaction was monitored by thin-layer chromatography by the disappearance of 4-nitrophthalonitrile (4NPN) spot. An inorganic precipitate was filtered off under reduced pressure and washed twice with solvent. To control the purity and concentration of the product in the solution, an aliquot of the DMAA solution was taken and poured into a fivefold excess of a mixture of water and hydrochloric acid in a ratio of 100:5 water, and the precipitate was filtered off and washed five times with hot water (80 °C). PNN was dried in a heating oven at 80 degrees for 24 h to constant weight. The solution was concentrated to 50 wt% of oligomer using a rotary evaporator. Yield was 99.5%.

^1^H NMR (600 MHz, DMSO): δ 3.42–4.00 (m, 2H, CH_2_), 6.47–8.04 (m, 6H, ArH).

Elemental analysis calculated: C 71.48, H 3.86, N 17.86; Found: 71.62, H 3.85, N 17.91.

### 2.4. Curing of PNN Oligomers with Novolac

**Figure 1:**
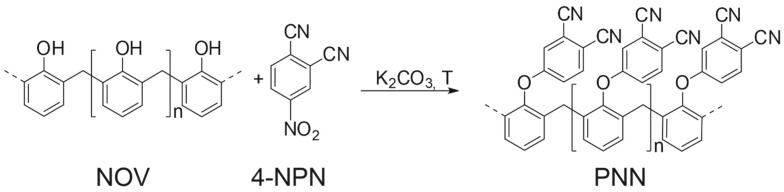
Scheme of PNN synthesis.

Dried PNN was combined with 25, 50 and 75 wt% of novolac (NOV) in a mortar (Figure 1). Mixtures containing PNN and XX wt% of NOV are named NOV XX respectively. The mixtures were melted at 150 °C for 5 min and cooled, after they were studied by DSC. 

Curing times were determined according to GOST 57779. PNN blends with different mass content of NOV were placed in a specially designed aluminium heating mold which was heated at 220 °C. Samples (0.5 ± 0.05 g) of the resins were placed in vials and put in the heating mold. At this moment the countdown was started. The resins were pressed with a thin steel probe every 10 s. Curing time was determined as a time to the moment of full solidification of the resin.

### 2.5. Preparation of Phthalonitrile Prepreg and Composite

42 g of DMAc and 42 g of NOV were added to 256 g of PNN solution in DMAc (50 wt%) and stirred on a magnetic stirrer. Prepreg sheets (30 × 30 cm, carbon or glass fibric) were prepared by applying the solution with a roller on carbon fabric sheets placed on an auxiliary film. Prepregs were left to dry for 12 h at room temperature and next dried in a vacuum bag at 110 °C, 3 h, 1 kPa; 10 layers of carbon prepreg (8 layers of glass prepreg) were plied in an aluminum mold, which was placed in Langzauner LZT-L 250 (Lambrechten, Austria) hot-press pre-heated to 220 °C. Composite molding was performed by hot pressing method following the curing program: 220 °C, 1.7 MPa, 20 min; 280 °C, 1.7 MPa, 30 min (heating rate 0.5 °C/min).

### 2.6. Flammability Tests

Flammability tests were performed in accordance with UL-94V. A gas burner was used for testing. The temperatures of the flame and of samples were controlled using thermocouples. To keep the sample in a flame of 1300 °C, the GFRP sample (20 × 5 × 0.2 cm^3^) was fixed with a laboratory stand. The flame was brought perpendicular to the GFRP surface, and a countdown was started. After 13 s the flame was removed and the time of residual burning and residual smoke emission time were recorded.

To hold for a minute in a flame of 900 °C, a GFRP sample was placed over a gas burner. After a minute had passed, the burner was removed, and the residual burning time and smoke emission time were recorded.

A sample (15 × 15 × 0.2 cm^3^) was fixed with a laboratory stand. Thermocouples were attached to the front and back of the sample in contact with the surface of the sample. The burner with the flame was brought perpendicular to the surface so the thermocouple was in the center of the flame. The experiment was carried out for 10 min, recording a change in temperature at the front and back sides of the samples.

## 3. Results and Discussion

### 3.1. Synthesis of PNN Oligomer

The first step of this work included an improvement of the procedure of PNN synthesis described in [62,64,65]. According the reported synthetic procedures, PNN oligomer was obtained from novolac phenolic resin by nucleophilic substitution of hydroxyl groups with 4-nitrophthalonitrile in DMF or NMP in the presence of potassium carbonate as a base (Figure 1). In several previous works [63,66], PNN was synthesized at high temperatures (80–100 °C) in non-quantitative yields. We suggested that this behavior was caused by formation of 4-hydroxyphthalonitrile as a side product in the presence of potassium nitrite and potassium carbonate [67,68,69]. At lower temperatures, the side reaction practically does not occur; however, the substitution reaction at room temperature also slows down, and the product yield is only 60% in 24 h [62]. Therefore, in this work, the reaction was carried out for 20 h but at 40 °C to reach full conversion of 4NPN into the desired product with a quantitative yield. In works [63,70,71], the full degree of substitution was not achieved and the only mention of 99% degree of substitution in PNN was found in only one work [62]. There, the substitution degree was defined by elemental analysis by evaluation of nitrogen content in the resin. In present work, the degree of substitution was controlled by ^1^H NMR. Due to the absence of wide singlets at 9.0–9.5 related to hydroxyl groups of novolac, we assumed that the reaction proceeded completely. The absence of signals at 8.4–9.0 related to 4-nitrophthalonitrile indicates a complete conversion of 4-nitrophthalonitrile.

**Figure 2:**
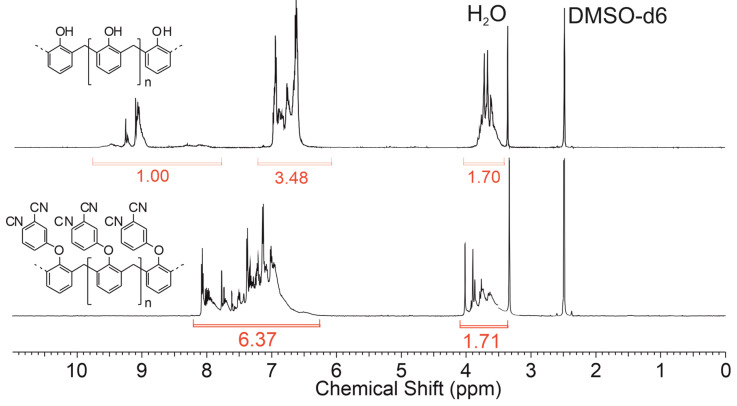
^1^H NMR spectrum of PNN synthesized in DMAc.

In the synthetic procedures described above, the reaction is carried out in high-boiling solvents such as NMP and DMF and includes several steps to separate the product [62,71]. To simplify isolation of the desired product from the reaction mixture, it was decided to use methylethylketone (MEK) as a solvent. This made it possible to filter the reaction mixture from inorganic fraction and then evaporate the solvent. However, the condensation reaction of MEK proceeded as a side reaction under selected conditions. This was evidenced by an indefinite number of signals in the aliphatic region at 0.5–2 ppm (Figure S1). The reaction proceeded the same way in acetone. Therefore, dimethylacetamide was used as the solvent to avoid side processes (Figure 2). 

**Figure 3:**
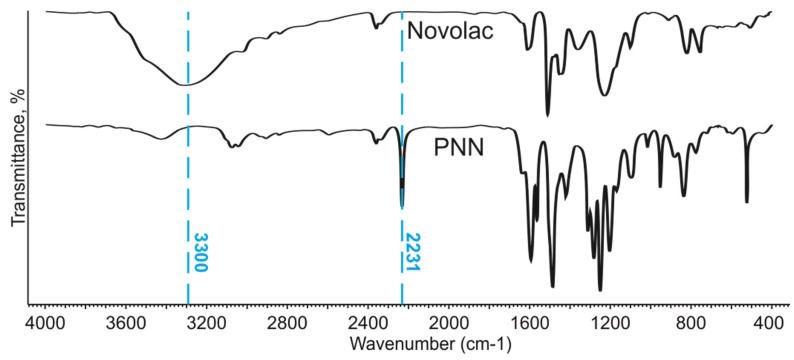
FT-IR of novolac and PNN.

FT-IR analysis of the synthesized PNN is shown in Figure 3. It can be seen that characteristic absorption band of cyano groups at 2231 cm^−1^ [72] was absent in pure novolac and appears after synthesis. The intensity of the characteristic absorption band of hydroxyl groups at 3300 cm^−1^ disappears after nucleophilic substitution at hydroxyls. 

There are no signals corresponding to 4-nitrophthalonitrile on the ^1^H NMR spectrum of PNN (600 MHz, DMSO-d6: d. 8.41–8.43 1H, d. 8.66–8.67 1H, s. 9.01 1H) (Figure 2). The characteristic absorption bands corresponding to NO_2_ (1538 and 1355 cm^−1^ [73]) are absent on the PNN spectrum (Figure 3) as additional evidence that 4-nitrophthalonitrile reacts completely during the synthesis.

### 3.2. Curing Behavior of PNN-NOV Blends

PNN with full substitution of hydroxyl groups could not be self-cured due to an absence of nucleophilic moieties needed for the curing reaction initiation [25,74,75,76,77]. To reach fast curing reaction and good miscibility of the resin components, it was decided to use a high content of neat novolac resin as curing initiator. Compositions of novolac with PNN with a novolac content of 25, 50 and 75 wt% were prepared for further studies. 

**Figure 4:**
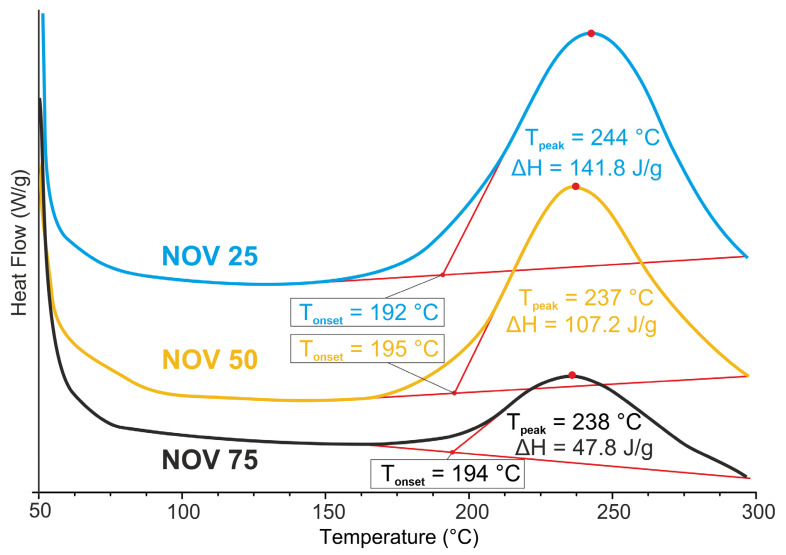
DSC of blends NOV 25, NOV 50, NOV 75.

The compositions were characterized by DSC (Figure 4). The onset temperature of polymerization was observed in the range of 192–195 °C for all three compositions. However, the heat release of the polymerization for NOV 75 was two to three times lower than for NOV 50 and NOV 25. This phenomenon was observed due to the ratio of hydroxyl groups (–OH) and phthalonitrile (–PN) groups: for NOV 75, the molar ratio of –OH to –PN groups was about 85:15 and thus heat release is in accordance with phthalonitrile content. For NOV 25, the molar ratio of –OH to –PN was 42:58, close to 1:1. This gives a base for suggestion that most reactive groups were involved in polymerization reaction, which increased the heat release of the curing reactions.

**Figure 5:**
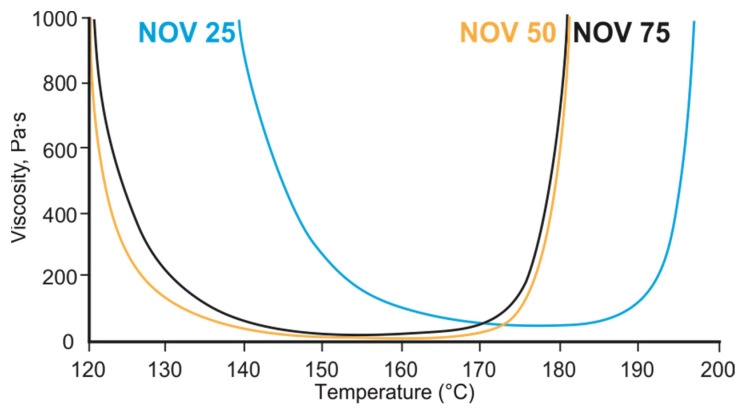
Viscosity–temperature profiles of blends NOV 25, NOV 50, NOV 75.

To estimate processability of the considered blends rheology study was performed. Viscosity (η)–temperature profiles were obtained for all the compositions (Figure 5). Viscosity values for the PNN-NOV system did not decrease below 10 Pa × s at 130–190 °C, which is higher than for most of the reported phthalonitrile resins in this temperature range (<1 Pa × s) [36,78]. For NOV 50 and NOV 75, the growth of viscosity related to a curing process was observed at 175 °C, while for NOV 25 it was shifted up to 190 °C. Viscosity growth onset temperature increased due to a lower number of initiating hydroxyls in NOV 25 causing lower polymerization rate. Based on the DSC and viscosity data, it was decided to carry out curing at 220 °C as all three compositions were guaranteed to polymerize at this temperature with a high rate. 

The mixtures were placed in an aluminum mold heated to 220 °C to estimate curing time (Table 1). The mixtures were pressed with a thin steel, poked every 10 s. The curing time was determined as a time to the moment of full solidification of the resin, when glass transition temperature exceeds 220 °C, which corresponds to a termination of fast polymerization. NOV 25 showed the shortest curing time, it is believed due to the ratio of phthalonitrile groups and hydroxyl groups being close to 1:1. Increasing the novolac content did not lead to an acceleration of the curing reaction, presumably due to the greater probability of formation of shorter oligomers after hydroxyl attack on phthalonitrile groups in the first polymerization phase rather than the formation of a cross-linked 3D network and the presence of more novolac resin, which is not able to cross-link itself with itself.

**Figure 6:**
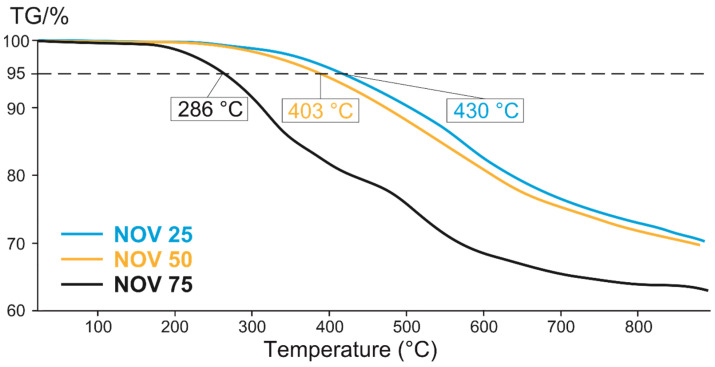
TGA curves for cured blends at 220 °C under nitrogen atmosphere.

The blends cured at 220 °C for 15 min were characterized by TGA under nitrogen atmosphere (Figure 6) to estimate their thermal stability. The most heat-resistant blend, NOV 25, had a 5% mass loss temperature of 430 °C, which also indicated a high degree of cross-linking in this thermoset which is in accordance with the results of curing time estimation and DSC experiments. The lowest T_5%_ of cured NOV 75 is explained by the high content of novolac resin, which decomposes in the range of 200–280 °C [79]. Based on the thermal performance and curing time experiments, NOV25 was chosen for composite fabrication.

### 3.3. FRP Manufacturing: Mechanical and Thermal Properties

**Figure 7:**
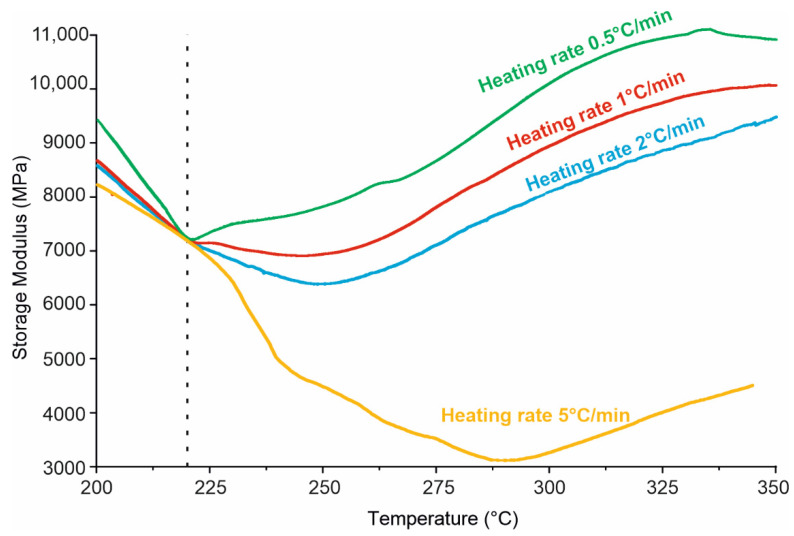
DMA of CFRP with NOV 25 (CFRP manufacturing temperature is 220 °C).

Based on viscosity, thermal properties and curing time, FRP were fabricated with the NOV 25 matrix by hot-pressing method. Carbon and glass fabric-reinforced composites were fabricated by impregnating each ply individually and assembling a preform for hot-pressing by [0]_n_ orientation. The resulting carbon fiber composite after pressing at 220 °C was studied by DMA at different heating rates of 5, 2, 1 and 0.5 °C/min after 220 °C (Figure 7, dashed line corresponds to 220 °C) to select a heating rate for free-standing post-curing. Softening of the matrix during post-curing should be avoided to maintain the shape of the part and the proper heating rate should be selected accordingly. The course of polymerization in the temperature range of 220–350 °C could be observed by changing the modulus of elasticity. It is assumed that polymerization proceeded as the modulus increased. At heating rates higher than 1 °C/min, the storage modulus did not grow before reaching 250 °C and witnessing a low polymerization rate, and at a rate of 0.5 °C/min it constantly increased. For further investigation, all composites were heated at a rate of 0.5 °C/min during post-curing.

Matrix weight loss and laminates glass transition temperatures were measured after post-curing with final temperatures 280 and 300 °C for 30 min, 1 h or 2 h (Table 2) (Figures S2–S4). As the post-curing temperature increases above 300 °C, the weight loss increases significantly and thus post-curing at 300 °C is considered as optimal for the processing. All the samples were dried at 130 °C before the first weighing to exclude the influence of water absorption occurred during sample preparation. Despite this, even a minimum post-curing time of 30 min at 280 °C led to a loss of 0.6 wt%, and 1 h was already more than 1 wt%. The loss of a large amount of matrix could significantly affect the mechanical characteristics of CFRPs due to the formation of additional porosity. Despite the increase in glass transition temperature with increasing post-curing time and temperature, it was decided to post-cure further CFRPs for 280 °C 30 min to minimize the effect on mechanical properties. Thus, the total processing time of CFRP fabrication was 2 h 50 min.

**Figure 8:**
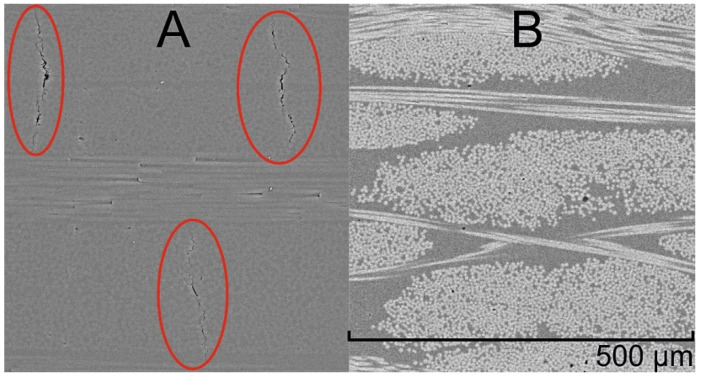
SEM microphotographs of CFRP (**A**), microcracks are circled in red, and GFRP (**B**) with NOV 25 as a matrix.

Mechanical properties of the composites with NOV 25 as a matrix were obtained and presented in the Table 3. In [50] by Nair et al., CFRPs were fabricated using partially phthalonitrile-substituted novolac resin as a matrix. Substitution degree was controlled by elemental analysis exclusively. At the same time, ILSS values measured for the composites did not exceed 20 MPa for all studied degrees of substitution. Due to the full degree of substitution and controlling the concentration of curing agent, it was possible to increase the ILSS in this work up to 26 MPa. SEM study of the carbon fabric composites revealed microcracking in transverse direction to the fabric. Presumably, the mechanical properties of the carbon fabric composites were affected by these microcracks formed during the post-curing (Figure 8A, circled in red). The matrix and carbon fabric shrank differently during cooling due to the difference in the thermal expansion coefficient, which led to the formation of microcracks. 

GFRP with a NOV 25 matrix were also obtained by the same molding method and at the same curing temperature. No microcracks were observed by SEM investigation of these composites (Figure 8B). Therefore, the mechanical properties could be expected to be higher than those of CFRPs despite the used reinforcement types. ILSS values of GFRP were higher, which indicated a better adhesion of the matrix to glass fabric, compared to carbon fabric, which was proved by the results of mechanical testing. Another explanation could be a better adhesion of hydroxyl-rich novolac-containing resins to glass fibers than to carbon fibers. To investigate this phenomenon resins with novolac concentrations of 20%, 15% and 5% were taken for GFRP fabrication and designated NOV20, NOV15 and NOV5, respectively. These blends were also cured at 220 °C and characterized by TGA to ensure their thermal stability (Figure S5) and DMA to confirm cross-linking occurrence. It is seen by these parameters that curing of NOV-20, NOV15 and NOV5 resulted in heat-resistant thermoset formation, indicating the required cross-linking degree. At the same time, all the mechanical properties of the composites decreased with a decrease in the novolac content. Thus, it was shown that NOV25 demonstrated the best behavior as a matrix for fast-curing glass-fiber composites among the studied blends, presumably due to a better adhesion between resin and fibers.

GFRP with quasi-isotropic ply orientation [0, ±45, 90]_2_ was fabricated using NOV 25 and tested on mechanical performance to eliminate an effect of ply orientation when comparing the properties of glass and carbon fiber composites. The studied CFRP were fabricated from symmetrical 2 × 2 twill carbon fabric, and as GFRP were reinforced with 8 × 3 satin fabric, quasi-isotropic ply orientation was thus needed to better compare ILSS values as an indicator of the interface strength. The results of mechanical testing performed at ambient conditions and at elevated temperatures are presented in Table 4. It is seen that quasi-isotropic plies orientation results in a decrease in mechanical properties of the composite in comparison to unidirectional composites tested at 0° direction. Compressive strength and ILSS values of the composites decreased gradually with testing temperature but remained at 82% at 250 °C while compressive strength remained at 71%, demonstrating good heat resistance of the composites. At the same time, it is seen that GFRP had higher ILSS values than CFRP which confirms the suggestion of better adhesion between the matrix and glass fibers then between the matrix and carbon fibers. 

Thus, it was shown that mechanical properties of the composites clearly depended on the matrix composition. NOV 25 appeared to be the optimal matrix composition providing the strongest interface between matrix and glass fibers resulting in the highest mechanical properties among the considered composites. Tensile strength of NOV 25 GFRP was 946 MPa, which is a bit higher than for the reported GFRP with phthalonitrile matrices [80,81,82] for which the highest value was 834 MPa [83]. The thermal and mechanical performance of the composites opens the perspective for applications in parts requiring operation at elevated temperatures up to 300 °C and as fire-protective walls in jet engines or e-vehicles.

### 3.4. Flammability Test

**Figure 9:**
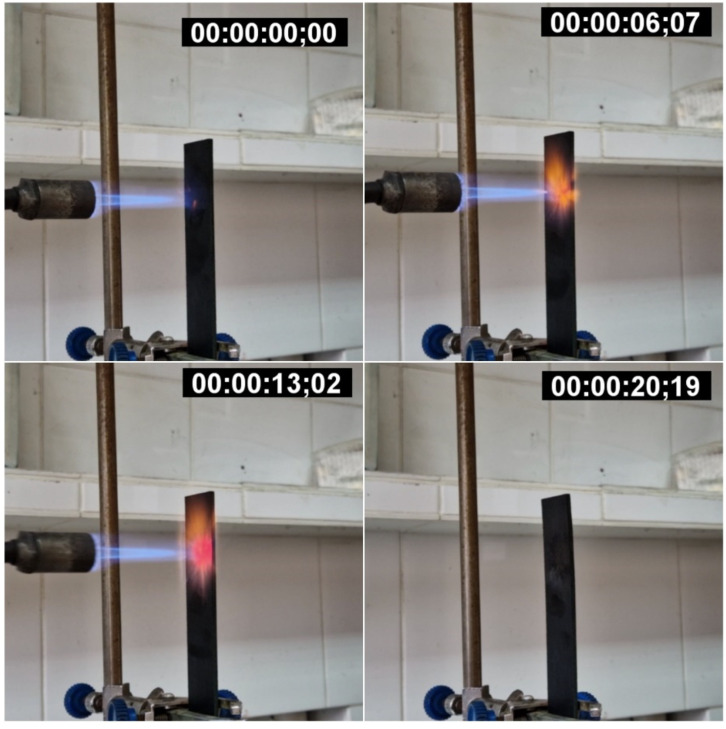
GFRP burning at 1300 °C for 13 s.

**Figure 10:**
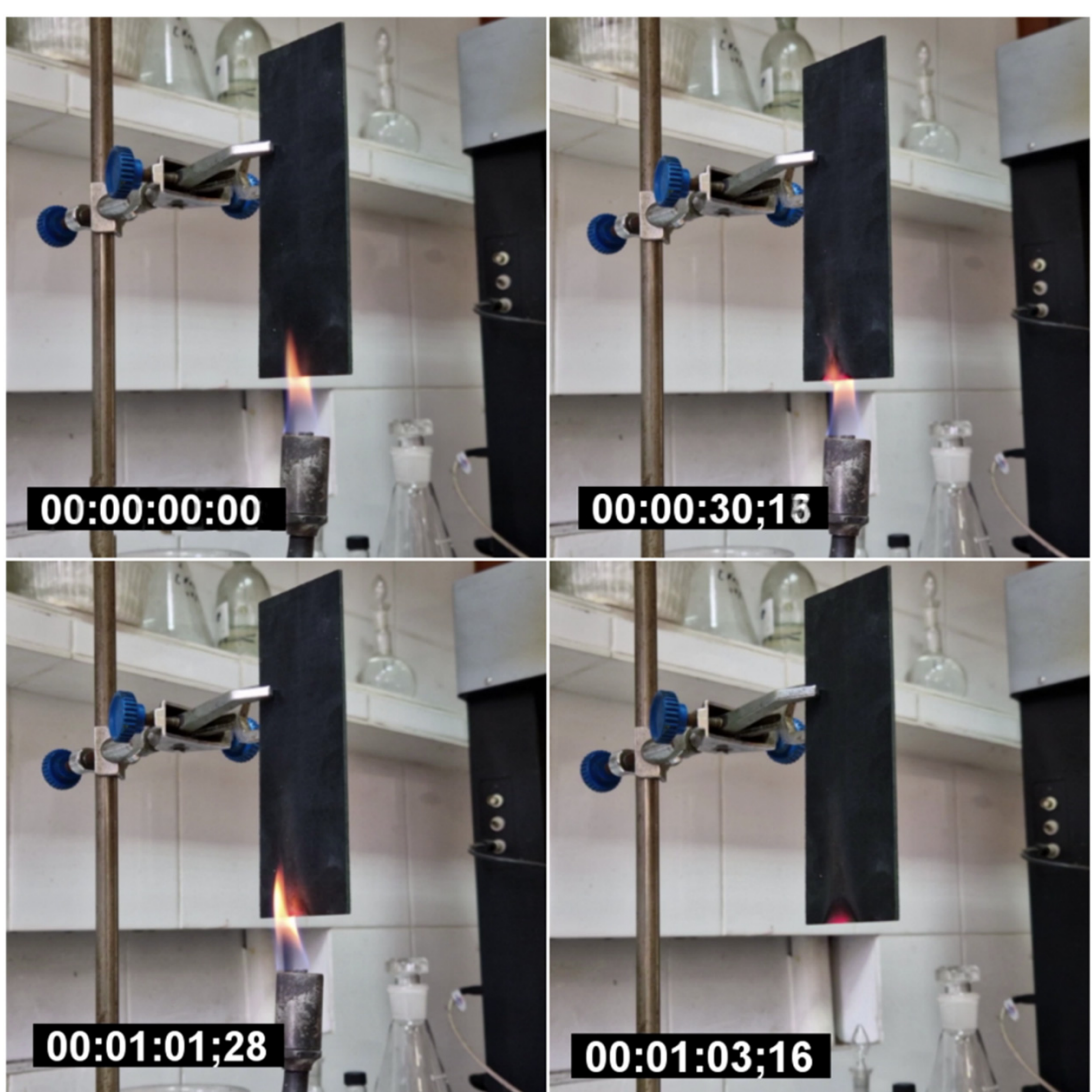
GFRP burning at 900 for 60 s.

GFRPs with a NOV 25 matrix were tested for flame retardance. Video of the experiment can be found in the Supplementary Materials. After 13 s of exposure to a flame at 1300 °C, the material instantly stopped burning, and smoke emission remained for 7 s (Figure 9) after removing the flame. When the material was kept in a colder flame (900 °C) for a minute, there was also no residual combustion after the burner was removed (Figure 10).

**Figure 11:**
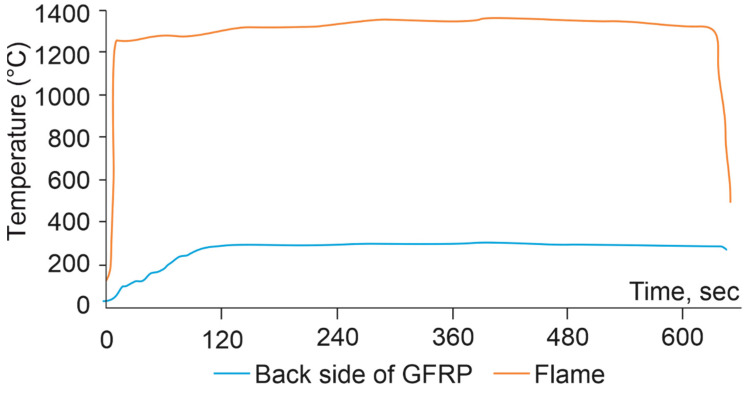
Dependence of the temperature of the back side of the GFRP during burning for 10 min at ~1300 °C.

An additional experiment holding the GFRP in a flame at 1300 °C for 10 min was carried out. Figure 11 shows the dependence of the temperature of the back side of the GFRP on time when exposed to a flame in the front side. The temperature of the back side did not rise above 300 °C. It is assumed that during the first 2 min of the experiment, the matrix burns out completely, since only during the first two minutes did the temperature of the back side grow and then reach the plateau. 

The results of the flammability tests demonstrated an incredible flame-retardant performance of the studied composites. According to UL94 classification, NOV 25 composites match the V-0 category. On the other hand, it is known that during combustion or pyrolysis of nitrile-containing resins evolution of HCN occurs [52,84,85]. At the same time the studies revealing that HCN oxidizes both in oxy-fuel (O_2_/CO_2_ atmosphere, [86]) and air-fired (O_2_/N_2_ atmosphere) conditions [87] were reported. This fact, along with the flammability behavior of the studied composite, makes it possible to conclude that NOV 25 GFRP can be considered as a flame-retardant material for application in the transport industries.

## 4. Conclusions

New fast-curing phthalonitrile resin for hot-pressing processing was developed based on PNN and novolac oligomers. The thermal and rheological behaviors of the blends with various novolac content were studied, and it was concluded that 25% of novolac weight fraction provided the best processing and operational characteristics for the resin for composites fabrication. NOV 25 cured at 220 °C was tested by TGA and the decomposition temperature (T_5%_) was found at 430 °C.

Carbon and glass fabric-reinforced composites were fabricated from solution-impregnated prepregs with 170 min curing cycle including 20 min pressing at 220 °C and free-standing post-curing at 280 °C, which is the lowest processing time reported for phthalonitriles. A post-curing heating rate of 0.5 °C/min was chosen based on DMA experiments to avoid devitrification of the matrix causing shape disturbances during complex-shaped part fabrication. Transversal microcracking caused by shrinkage and the difference between thermal expansion coefficients of the matrix and the fibers was found in CFRP. Despite this, carbon fabric composites demonstrated ILSS values of 26.7 MPa, which are higher than ILSS values reported for composites of such type. Outstanding mechanical characteristics were observed for GFRP with ILSS above 86 MPa for [0]-plied composites and 37 MPa for quasi-isotropic laminate. GFPR retained 82% of ILSS values at 250 °C and 71% of compressive strength. 

Based on flammability test results, the composites were classified as V-0 according to the UL94 ratings. Thus, it was shown that the presented GFRP can be applied in hot zones up to operating temperatures of 250 °C. In combination with its flame retardancy, this laminate is prospective for the manufacturing of fire barriers in the aircraft industry as well as of battery cases for electric vehicles.

## Figures and Tables

**Table 1:** Curing time of blends at 220 °C.

Blend	Curing Time at 220 °C, min
NOV 25	5
NOV 50	7
NOV 75	12

**Table 2:** Selecting of post-curing mode.

	Post-Curing Temperature, °C	Post-Curing Time		
		30 min	1 h	2 h
T_g_, °C	280	329	324	354
	300	360	372	379
Weight loss of matrix, wt%	280	0.6	1.3	1.4
	300	2.2	2.9	3.9

**Table 3:** Mechanical properties of FRPs plied with [0]_n_ orientation at 0° direction.

Fiber	Carbon	Glass	Glass		
Matrix	NOV25		NOV20	NOV15	NOV5
Interlaminar shear strength τ_13_, MPa	26.7 ± 1.1	86.2 ± 2.9	78.6 ± 2.8	72.0 ± 4.7	66.4 ± 4.0
Compressive strength σ_11−_, MPa	533 ± 67	620 ± 49	555 ± 32	496 ± 35	462 ± 27
Compressive modulus, GPa	60.5 ± 6.6	34.4 ± 1.5	33.3 ± 2.6	33.6 ± 3.8	27.4 ± 3.6
Flexural strength, MPa	632 ± 33	946 ± 28	797 ± 30	906 ± 16	866 ± 70
Tensile strength, σ_11+_, MPa	728 ± 35	698 ± 19	599 ± 26	610 ± 24	593 ± 19
Tensile modulus, E_11+_, GPa	51.8 ± 2.8	35.7 ± 0.5	32.1 ± 0.8	32.0 ± 0.5	31.0 ± 1.0
T_g_, °C	345	308	301	309	307

**Table 4:** Mechanical properties of quasi-isotropic FRP with T10 and NOV as a matrix.

Measurement Temperature, °C	RT	200	250
Interlaminar shear strength τ_13_, MPa	37.6 ± 1.6	33.1 ± 3.0	30.7 ± 1.7
Compressive strength σ_11−_, MPa	285.6 ± 5.6	207.2 ± 6.1	203.7 ± 6.7
Compressive modulus, GPa	21.2 ± 0.5	16.3 ± 0.5	16.8 ± 0.8

## Data Availability

The data presented in this study are available on request from the corresponding author.

## Supplementary Materials

The following supporting information can be downloaded at https://www.mdpi.com/article/10.3390/polym14224975/s1: Figure S1: 1H NMR spectrum of PNN synthesized in MEK; Figure S2: DMA of CFRP post-cured at 280 °C for 30 min, 1 h and 2 h; Figure S3: DMA of CFRP post-cured at 300 °C for 30 min, 1 h and 2 h; Figure S4: DMA of GFRP with NOV 25 post-cured at 280 °C for 30 min; Figure S5: TGA curves for cured blends at 220 °C under nitrogen atmosphere; Figure S6: GFRP after burning for 10 min at 1300 °C; Video S1: Burning for 13 s at 1300 °C; Video S2: Burning for 60 s at 900 °C; Video S3: Burning for 10 min at 1300 °C.
