# Supplementary material for: Fast-Processable Non-Flammable Phthalonitrile-Modified Novolac/Carbon and Glass Fiber Composites
Source: Polymers (Basel). 2022 Nov 17;14(22):4975. doi: 10.3390/polym14224975 (PMC9699085; doi:10.3390/polym14224975)
Supplement: Supplementary file 1 [file polymers-14-04975-s001.zip › Supplementary_revised.docx]

**Supplementary application for**

**«Fast-processable non-flammable phthalonitrile modified novolac/carbon and glass fiber composites»**

Daria I. Poliakova^1^, Oleg S. Morozov^1^, Ekaterina A. Afanaseva^1^, Вoris A. Bulgakov*^1^, Alexander V. Babkin^1^, Alexey V. Kepman^1^, Viktor V. Avdeev^1^

1. M.V. Lomonosov Moscow State University, Department of Chemistry, Division of Chemical Technology and New Materials, 119991, Leninskie gory st, 1-11, Moscow, Russia

**Table of contents**

[Figure S1. ^1^H NMR spectrum of PNN synthesized in MEK 2](#_Toc119073986)

[Figure S2. DMA of CFRP postcured at 280 ⁰C for 30 min, 1 hour and 2 hours. 2](#_Toc119073987)

[Figure S3. DMA of CFRP postcured at 300 ⁰C for 30 min, 1 hour and 2 hours. 3](#_Toc119073988)

[Figure S4. DMA of GFRP with NOV 25 postcured at 280 ⁰C for 30 min 3](#_Toc119073989)

[Figure S5. TGA curves for cured blends at 220 °C under nitrogen atmosphere 4](#_Toc119073990)

[Figure S6. GFRP after burning for 10 minutes at 1300C. 4](#_Toc119073991)





# Figure S1. ^1^H NMR spectrum of PNN synthesized in MEK


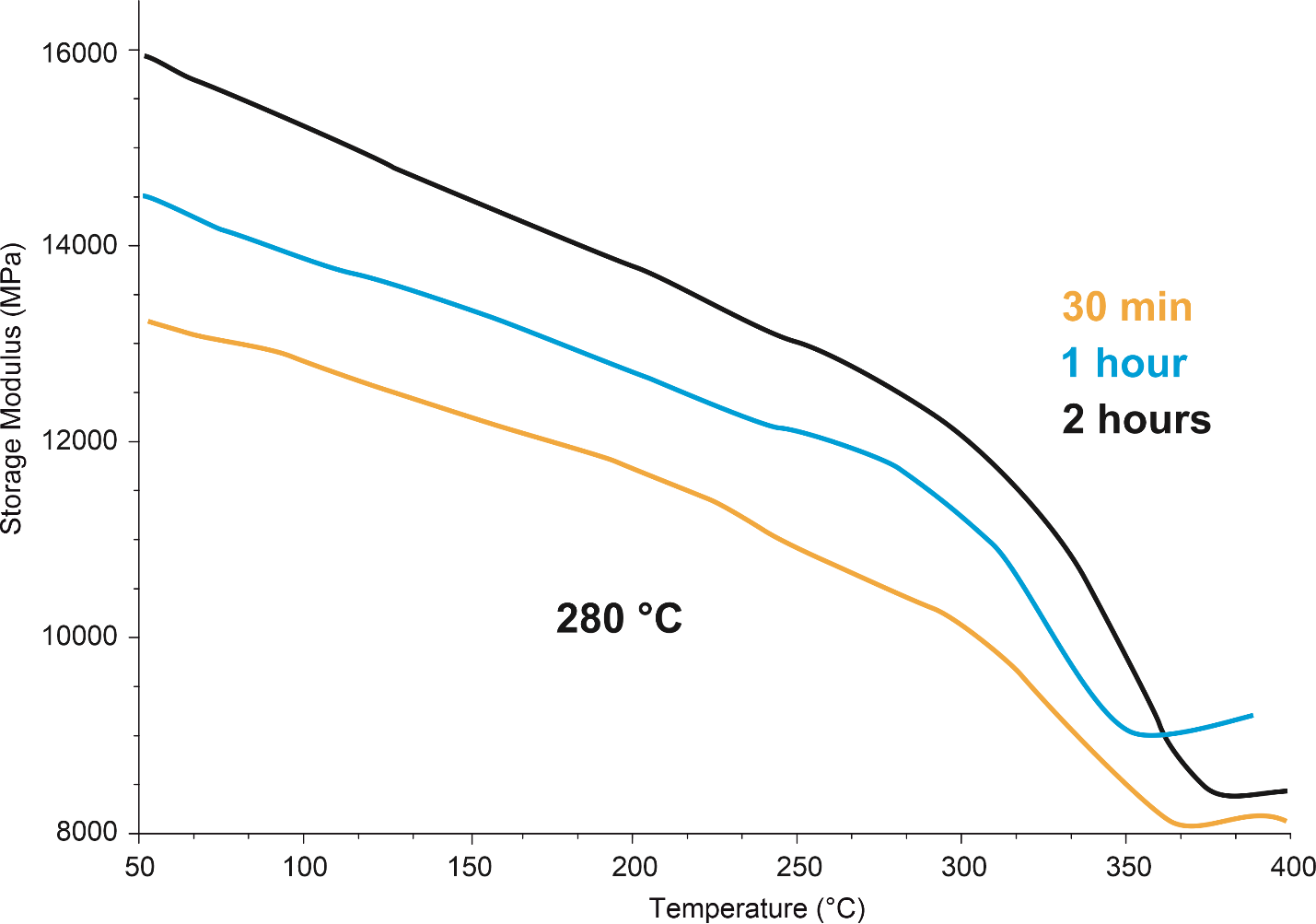


# Figure S2. DMA of CFRP postcured at 280 ⁰C for 30 min, 1 hour and 2 hours.


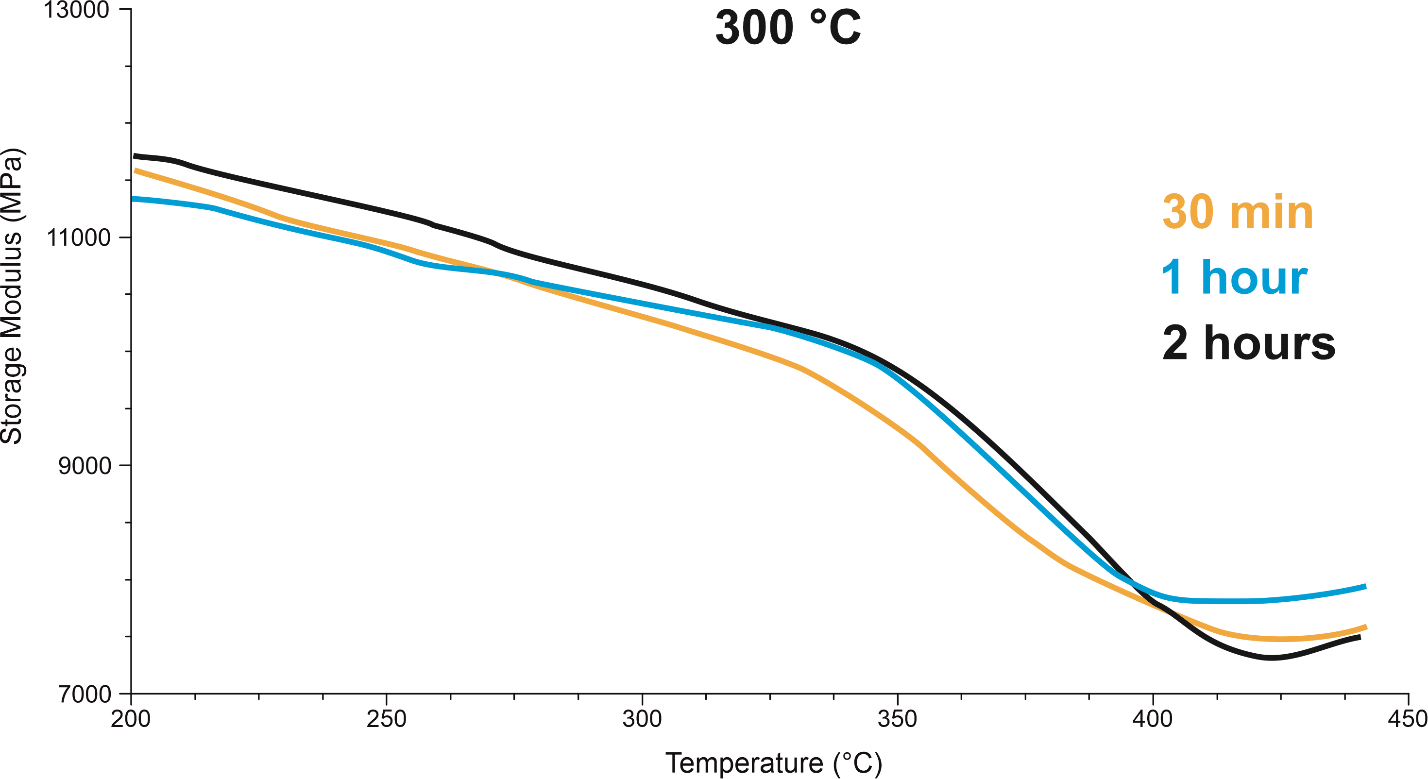


# Figure S3. DMA of CFRP postcured at 300 ⁰C for 30 min, 1 hour and 2 hours.


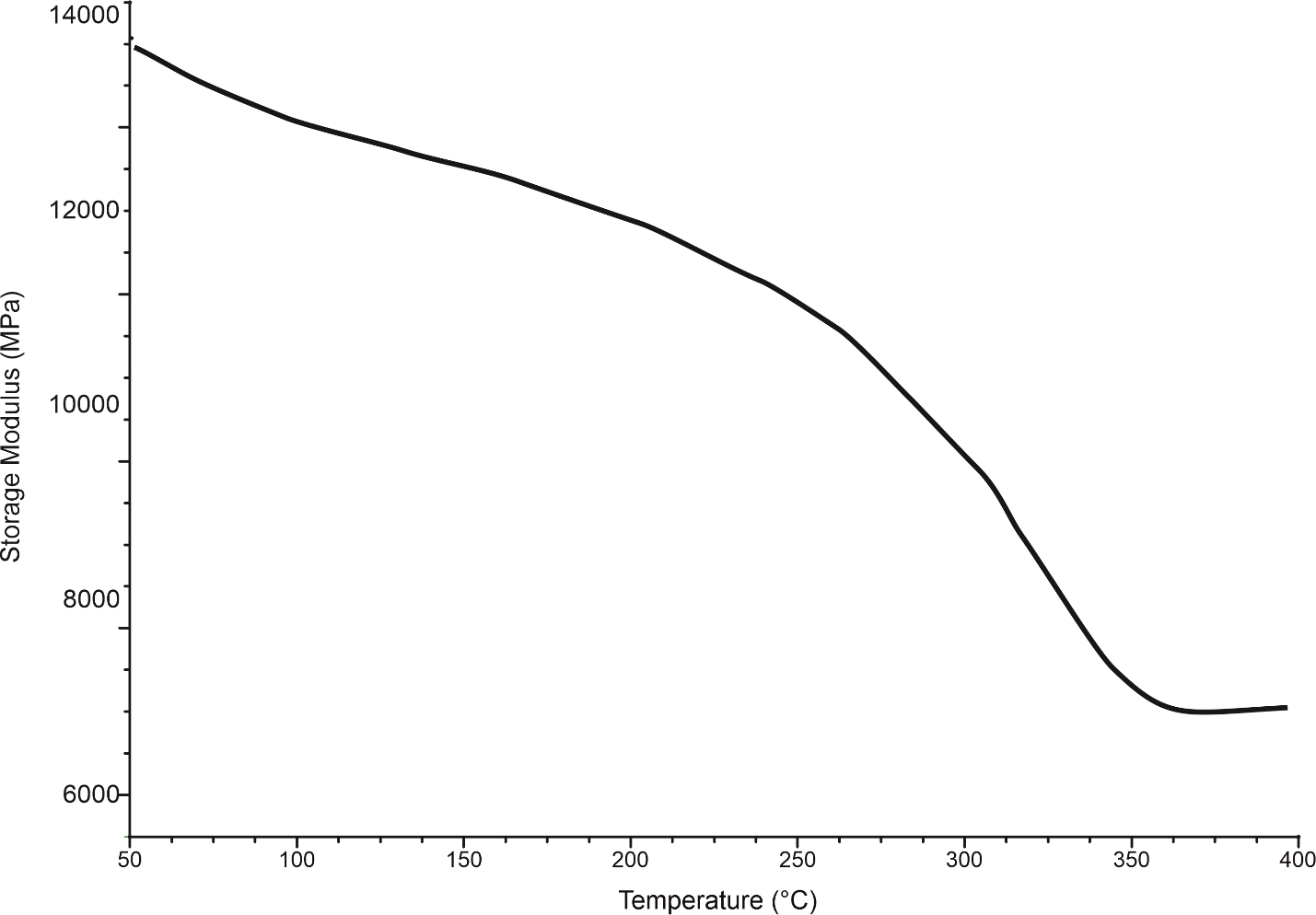


# Figure S4. DMA of GFRP with NOV 25 postcured at 280 ⁰C for 30 min


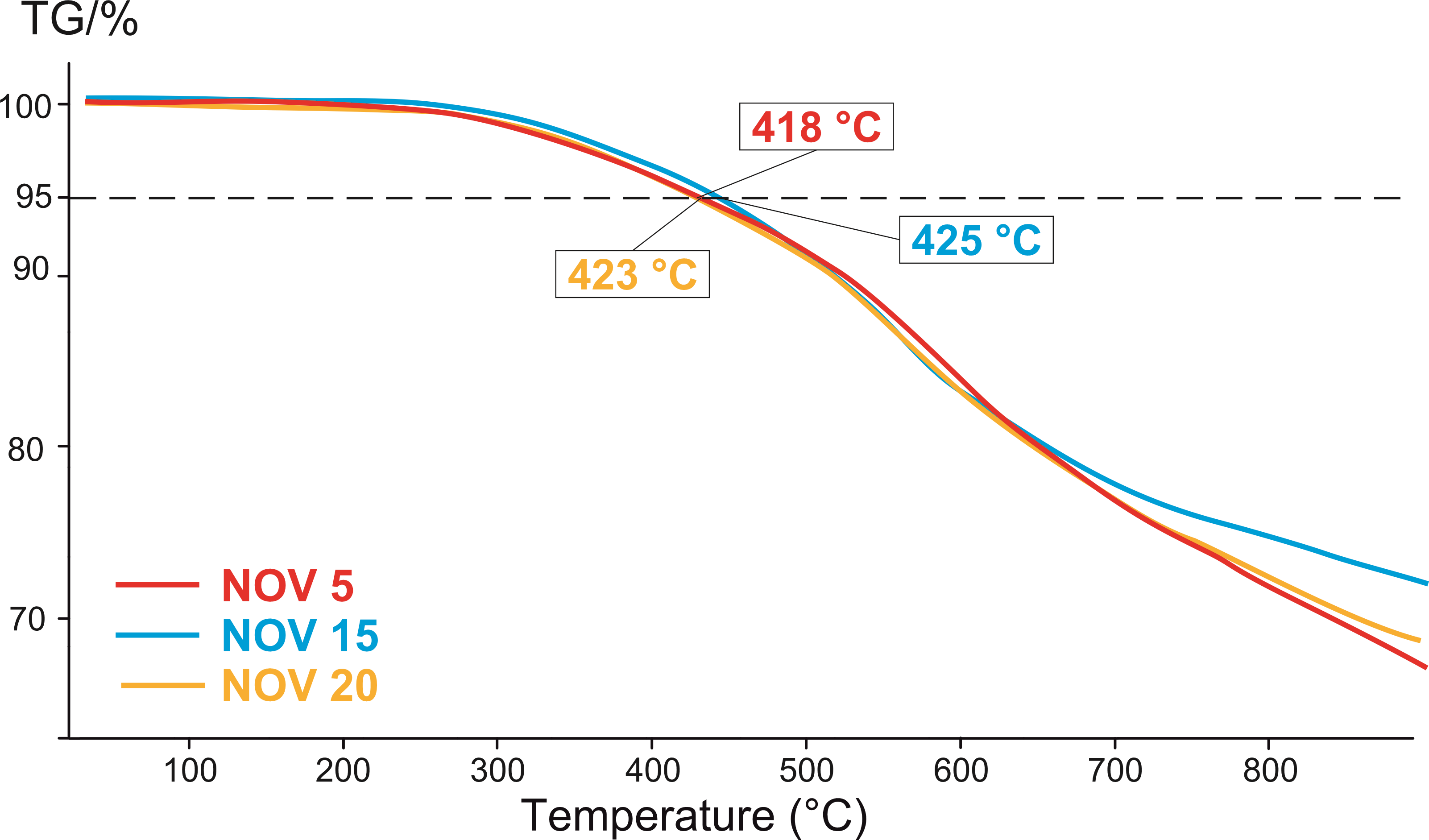


# Figure S5. TGA curves for cured blends at 220 °C under nitrogen atmosphere


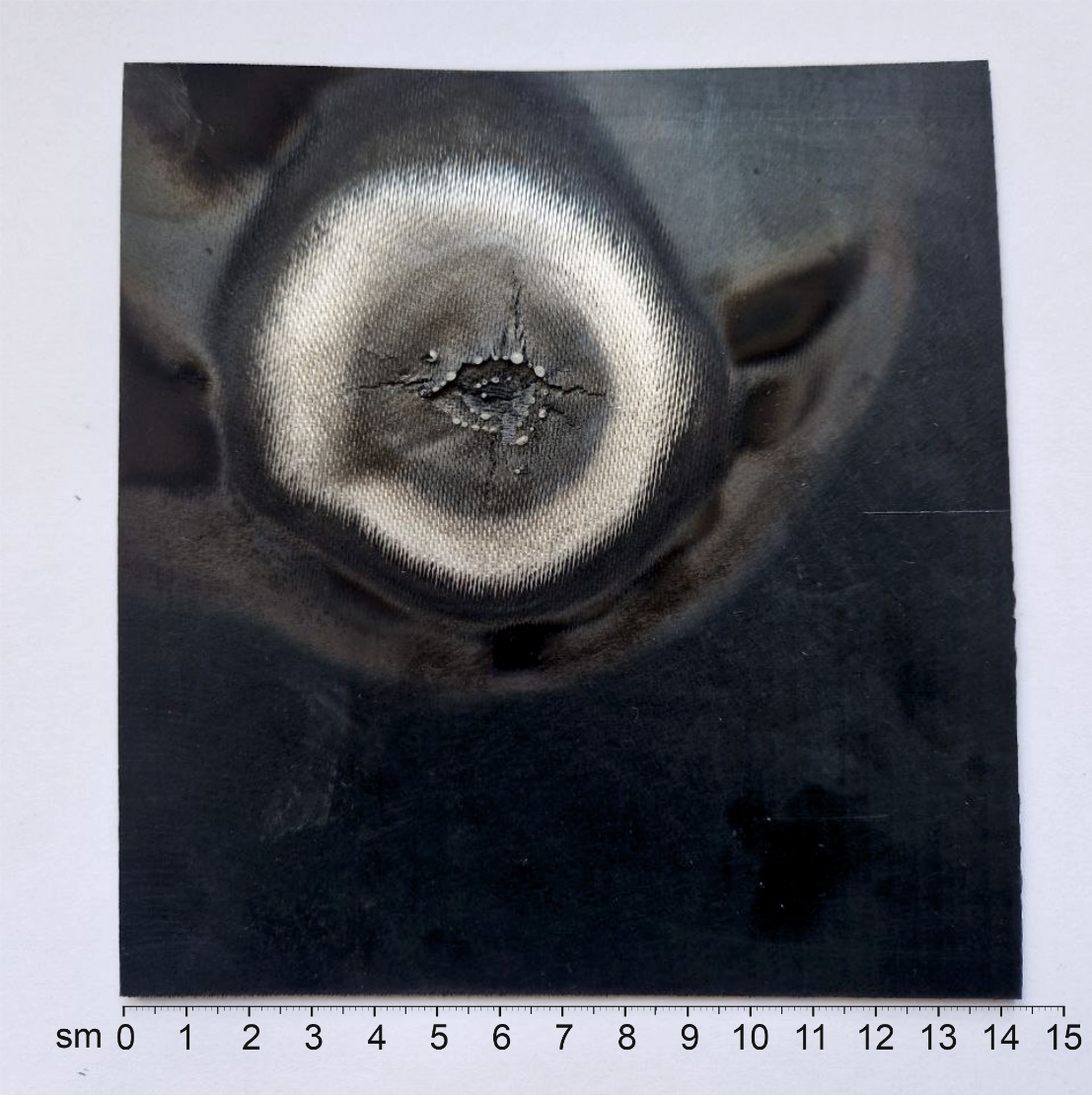


# Figure S6. GFRP after burning for 10 minutes at 1300C.
